# Optimal stochastic power flow using enhanced multi-objective mayfly algorithm

**DOI:** 10.1016/j.heliyon.2024.e26427

**Published:** 2024-02-18

**Authors:** Jianjun Zhu, Yongquan Zhou, Yuanfei Wei, Qifang Luo, Huajuan Huang

**Affiliations:** a College of Artificial Intelligence, Guangxi University for Nationalities, Nanning, 530006, China; b Faculty of Information Science and Technology, Universiti Kebangsaan Malaysia, Bangi, 43600, Selangor, Malaysia; c Xiangsihu College of Gunagxi University for Nationalities, Nanning, Guangxi, 532100, China; d Guangxi Key Laboratories of Hybrid Computation and IC Design Analysis, Nanning, 530006, China

**Keywords:** Multi-objective mayfly algorithm, Stochastic power flow, Renewable energy, Metaheuristic

## Abstract

For the classical multi-objective optimal power flow (MOOPF) problem, only traditional thermal power generators are used in power systems. However, there is an increasing interest in renewable energy sources and the MOOPF problem using wind and solar energy has been raised to replace part of the thermal generators in the system with wind turbines and solar photovoltaics (PV) generators. The optimization objectives of MOOPF with renewable energy sources vary with the study case. They are mainly a combination of 2–4 objectives from fuel cost, emissions, power loss and voltage deviation (VD). In addition, reasonable prediction of renewable power is a major difficulty due to the discontinuous, disordered and unstable nature of renewable energy. In this paper, the Weibull probability distribution function (PDF) and lognormal PDF are applied to evaluate the available wind and available solar power, respectively. In this paper, an enhanced multi-objective mayfly algorithm (NSMA-SF) based on non-dominated sorting and the superiority of feasible solutions is implemented to tackle the MOOPF problem with wind and solar energy. The algorithm NSMA-SF is applied to the modified IEEE-30 and standard IEEE-57 bus test systems. The simulation results are analyzed and compared with the recently reported MOOPF results.

## 1 Introduction

The optimal power flow (OPF) problem was first solved by an optimization method with exact second order derivatives in 1984 [1], and its definition is network constrained economic OPF was provided in Ref. [2]. The purpose of the classical OPF problem is to optimize a certain objective or multi-objective of minimizing fuel cost, emission, real power loss, VD, etc. While minimizing single or multi-objectives, feasible control variables are calculated to satisfy various equality and inequality constraints. However, with the large-scale use of generators in thermal power plants, large amounts of harmful emissions are released, thus causing great harm to the environment [3]. Emissions are also universally considered as a necessary goal to reduce environmental pollution. Hence, MOOPF problem consists of fuel cost, emission, power loss and VD in this study. Due to the highly nonlinear nature of the MOOPF and the presence of generally conflicting objectives, its solution requires the use of efficient techniques.

The weighted sum approach is a well-known method for solving MOOPF, that each objective is assigned a pre-defined weighting factor. But the weighting factor is determined after a significant amount of experimentation and needs to judge the obtained results of objectives. This approach aims to multiply multiple objective functions with different weights and then cumulatively combine them into a single objective function for better optimization and solution. Furthermore, solving the MOOPF problem with this strategy requires a lot of computational time [4]. Some recent articles that use a combination of weighting and metaheuristic algorithms, such as, differential evolution (DE) [5], differential search algorithm (DSA) [6], moth-swarm algorithm (MSA) [7]. The multi-objective metaheuristic algorithm solves problems by obtaining the Pareto front (PF). In the present study of MOOPF, MOEA/D was applied to resolve MOOPF in Ref. [8], where the penalty function approach is combined to tackle the constraints. The internal search algorithm (ISA) [9] and differential evolution (ESDE-MC) [10] were also applied to resolve the MOOPF problem.

The large-scale utilization of fossil energy as fuel in conventional thermal power generation units emits large amounts of harmful greenhouse gases into the environment, which leads to environmental pollution and climate degradation. In response to the above problems, the MOOPF with renewable energy is proposed, which is to use renewable energy power generators in the power generation system to replace traditional thermal generators. However, the reasonable prediction of renewable energy is a complex and difficult problem. Affected by wind speed changes, wind turbines may cause voltage fluctuation and flicker [11]. In Ref. [12], the fitness-distance balance-based stochastic fractal search (FDB-SFS) is proposed to solve the single objective OPF problem for multi-terminal direct current transmission lines combining both renewable energy sources and voltage source converter-based. The concept of Downward Risk (DR) was proposed to study the POPF of wind power integration, which manifests as the semi-variance of power generation costs in an uncertain environment of wind power [13]. In Ref. [14], the OPF problem with thermal, wind, solar, small hydro, and tidal energy is solved by an improved stochastic fractal search algorithm. In Ref. [15], Weibull PDF was used to fit the alternating properties of wind farms (WF). In Ref. [16] presented a dynamic economic dispatch (DED) model for large-scale wind power penetration. In Ref. [17] indicates a complex correlation between the line dynamic thermal rating (DTR) and the WF power output, and use the analytical PDF and CDF formulas to calculate the actual output.

In a modification of the IEEE-30 bus system, wind-driven generations and solar PV generations are combined in the power system. Specifically, in the modified test system, three thermal generators were replaced by two wind turbines and one photovoltaic generator, respectively. The Weibull and log-normal PDF are used for calculating available wind and solar energy, respectively. The computational complexity of the MOOPF problem with stochastic wind and solar power is greatly increased as a result of the discontinuous, disordered and unstable properties of renewable energy sources [18]. To solve this problem, an improved multi-objective mayfly algorithm NSMA-SF is proposed, which adopts a constraint processing technique named superiority of feasible solutions (SF). The simulation results show that NSMA-SF can provide an effective compromise for MOOPF. The main contributions can be outlined as follows.

● An improved multi-objective mayfly algorithm (NSMA-SF) is proposed. In NSMA-SF, three strategies are incorporated to enhance the capabilities of the basic mayfly algorithm.
● The enhanced mayfly algorithm to solve the MOOPF problem with wind and solar energy on two different bus systems, and the robustness, superiority and efficiency of the algorithm are demonstrated.
● The experiment results show that the proposed NSMA-SF can be an effective for MOOPF problem.

The rest of study is organized as follows. The mathematical model of MOOPF with stochastic renewable power is introduced in Section 2. Mathematical models of wind and solar power generators are described in Section 3. In Section 4, the multi-objective algorithm NSMA-SF is proposed for MOOPF with wind and solar energy. Section 5 shows the simulation results and comparative studies. The conclusion and future work are presented in Section 6.

## 2 The MOOPF problem with renewable energy sources model

Mathematically, the MOOPF consists mainly of the selected optimization objective $f_{i}\left(\bar{x},\bar{y}\right)$, control and state variables ($\bar{x}$ and $\bar{y}$) and constraints ($g\left(\bar{x},\bar{y}\right)$ and $h\left(\bar{x},\bar{y}\right)$), which can be formulated as Eq. (1).

(1) $$\text{Minimize}\;F\left(\bar{x},\bar{y}\right)=\left\{f_{1}\left(\bar{x},\bar{y}\right),f_{2}\left(\bar{x},\bar{y}\right),...,f_{M}\left(\bar{x},\bar{y}\right)\right\}$$

Subject to $g\left(\bar{x},\bar{y}\right)\leq 0$.

$$h\left(\bar{x},\bar{y}\right)=0$$

### 2.1 Control variables and state variables

The MOOPF problem with wind and solar energy includes the control (or independent) variables, which is composed of the active power, voltage magnitude, and shunt compensators. The control variable $\bar{x}$ is represented in the vector form as Eq. (2):

(2) $$\bar{x}=\left[P_{G_{B2}},...,P_{G_{BNG}},V_{G_{B1}},...,V_{G_{BNG}},Q_{C_{1}},...,Q_{C_{NC}}\right]$$

where $P_{G_{Bi}}$ is the active power of the generator at the *i*-th bus (except the slack generator $P_{G_{B1}}$), $V_{G_{Bi}}$ denotes the voltage magnitude, *Q*_*Ck*_ represents the shunt compensation at *k*-th bus.

The state variable $\bar{y}$ represents a quantitative description of the actual state of the entire power system network. The state (or dependent) variable *y* is defined in vector form as Eq. (3):

(3) $$\bar{y}=\left[P_{G_{B1}},V_{L_{B1}},...,V_{L_{BNL}},Q_{G_{1}},...,Q_{G_{NG}},S_{l_{1}},...,S_{l_{nl}}\right]$$

where *V*_*LBi*_ denotes the actual voltage at *i*-th bus. The reactive power of the generator is written in *Q*_*G*_, *S*_*l*_ represents the loading of the transmission line.

### 2.2 Constraints

Constraints are classified as equality constraints and inequality constraints. Specifically, equality constraints serve primarily to regulate the equilibrium of power input and output, whereas operational constraints give rise to the inequality constraints.

#### 2.2.1 Equality constraints

In a power network, the actual active power value of the generator is as large as the reactive power output. The equality constraints formulated as Eqs. (4), (5):

(4) $$P_{G_{Bi}}-P_{D_{Bi}}-V_{Bi}\sum_{j=1}^{NB}V_{Bj}\left[G_{Bij}\;\cos\left(\theta_{Bij}\right)+B_{Bij}\;\sin\left(\theta_{Bij}\right)\right]=0$$

(5) $$Q_{G_{Bi}}-Q_{D_{Bi}}-V_{Bi}\sum_{j=1}^{NB}V_{Bj}\left[G_{Bij}\;\cos\left(\theta_{Bij}\right)+B_{Bij}\;\sin\left(\theta_{Bij}\right)\right]=0$$

where $\theta_{Bij}$ denotes the angle difference between the voltage phasor values on the *i*-th and *j*-th bus, *NB* indicates the number of total buses, $P_{D_{Bi}}$ is the power demanded of the generator, $Q_{D_{Bi}}$ represents the reactive power demanded of the generator, *G*_*Bij*_ and *B*_*Bij*_ are identified as the transfer conductance and susceptance values between *i-*th and *j-*th bus.

#### 2.2.2 Inequality constraints

Inequality constraints are based on inequality constraints caused by equipment operating constraints can be defined as Eqs. (6), (7), (8), (9), (10), (11), (12).

(a) Generator constraints:

(6) $$P_{G_{Bi}}^{\min}\leq P_{G_{Bi}}\leq P_{G_{Bi}}^{\max},\forall i\in NG$$

(7) $$V_{G_{Bi}}^{\min}\leq V_{G_{Bi}}\leq V_{G_{Bi}}^{m\text{ax}},\forall i\in NG$$

(8) $$Q_{\text{Gi}}^{\min}\leq Q_{Gi}\leq Q_{\text{Gi}}^{\max},\forall i\in NG$$

(b) Transformer constraints:

(9) $$T_{j}^{\min}\leq T_{j}\leq T_{j}^{m\text{ax}},\forall j\in NT$$

(c) Shunt compensator constraints:

(10) $$Q_{\text{Ck}}^{\min}\leq Q_{Ck}\leq Q_{Ck}^{\max},\forall k\in NC$$

(d) Security constraints:

(11) $$V_{\text{Lp}}^{\min}\leq V_{\text{Lp}}\leq V_{\text{Lp}}^{\max},\forall p\in NL$$

(12) $$S_{\text{lq}}^{\min}\leq S_{\text{lq}}\leq S_{\text{lq}}^{\max},\forall q\in nl$$

### 2.3 Optimization objectives and case studies

#### 2.3.1 Generation cost

In the MOOPF, the minimization of the generation cost is the main objective. The generation cost is defined as the following (13), (14):

(13) $$\text{Cost}=\sum_{i=1}^{\text{NG}}\left(a_{i}+b_{i}P_{G_{Bi}}+c_{i}P_{G_{Bi}}^{2}+d_{i}E_{G_{Bi}}\right)$$

(14) $$E_{Gi}=\left|\sin\left(e_{i}\cdot \left(P_{G_{Bi}}^{\max}-P_{G_{Bi}}^{\min}\right)\right)\right|$$

where *E*_*Gi*_ denotes the energy cost of valve point loading effect in traditional thermal power generators, *a*_*i*_, *b*_*i*_ and *c*_*i*_ denote the corresponding generator cost coefficients. *d*_*i*_ and *e*_*i*_ indicate the valve load effect coefficients. The specific values of each coefficients can be presented in Table 1.

**Table 1:** Cost coefficients of thermal generator in two test systems.

Network	Generator no.	Bus	*a* ($/h)	*b* ($/MWh)	*c* ($/MW^2^h)	*d* ($/h)	*e* (rad/MW)
IEEE-30 bus	G1	1	0	2.00	0.0038	18	0.037
	G2	2	0	1.75	0.0175	16	0.038
	G8	8	0	3.25	0.0083	12	0.045
IEEE-57 bus	G1	1	0	20	0.07758	18	0.037
	G2	2	0	40	0.01000	16	0.038
	G3	3	0	20	0.25000	13.5	0.041
	G6	6	0	40	0.01000	18	0.037
	G8	8	0	20	0.02222	14	0.04
	G9	9	0	40	0.01000	15	0.039
	G12	12	0	20	0.03230	12	0.045

#### 2.3.2 Emission

With the increasingly stringent environmental protection requirements, reducing emissions is a reasonable optimization objective. Conventional thermal power generation will emit a large amount of greenhouse and harmful gases. The emission is calculated as Eq. (15), the specific values of emission coefficients are presented in Table 2.

(15) $$\text{Emission}=\sum_{i=1}^{\text{NG}}\left[\left(\alpha_{i}+\beta_{i}P_{Gi}+\gamma_{i}P_{Gi}^{2}\right)+\varpi_{i}e^{\left(\mu_{i}P_{Gi}\right)}\right]$$

**Table 2:** Emission coefficients of thermal generator in two test systems.

Network	Generator no.	Bus	*α*	*β*	*γ*	*ω*	*μ*
IEEE-30 bus	G1	1	0.0409	−0.056	0.0649	0.0002	6.667
	G2	2	0.0254	−0.06	0.0564	0.0005	3.333
	G3	8	0.0533	−0.036	0.0338	0.002	2.000
IEEE-57 bus	G1	1	0.04	−0.05	0.06	0.00002	0.5
	G2	2	0.03	−0.06	0.05	0.00005	1.5
	G3	3	0.04	−0.05	0.04	0.00001	1.0
	G6	6	0.035	−0.03	0.035	0.00002	0.5
	G8	8	0.05	−0.05	0.045	0.00004	2.0
	G9	9	0.045	−0.04	0.05	0.00001	2.0
	G12	12	0.06	−0.05	0.05	0.00001	1.5

#### 2.3.3 Real power loss

In the transmission of a real power system, the power loss (or outages) in the system cannot be avoided, and power loss can be calculated as Eq. (16):

(16) $$\text{Loss}=\sum_{i=1}^{\text{NG}}G_{ij}\left[\left(V_{Bi}^{2}+V_{Bj}^{2}-2V_{Bi}V_{Bj}\;\cos\left(\theta_{Bij}\right)\right)\right]$$

#### 2.3.4 Voltage deviation (VD)

For a power system, the VD is a significant indicator to detect the voltage quality. The total VD is formulated as Eq. (17):

(17) $$\text{VD}=\left(\sum_{p=1}^{\text{NL}}\left|V_{LP}-1\right|\right)$$

### 2.4 Case studies

In this paper, 10 different multi-objective case studies are conducted in the modified IEEE-30 and the classic IEEE-57 bus system. The multi-objective of the MOOPF problem is composed of two to four objectives. Table 3 summarizes the study cases.

**Table 3:** A summary of case studies.

IEEE 30-bus system					IEEE 57-bus system				
Case no.	Cost	Emission	Power loss	VD	Case no.	Cost	Emission	Power loss	VD
Case 1	**✓**	**✓**			Case 7	**✓**	**✓**		
Case 2	**✓**			**✓**	Case 8	**✓**			**✓**
Case 3	**✓**	**✓**	**✓**		Case 9	**✓**		**✓**	**✓**
Case 4	**✓**	**✓**		**✓**	Case 10	**✓**	**✓**	**✓**	**✓**
Case 5	**✓**		**✓**	**✓**					
Case 6	**✓**	**✓**	**✓**	**✓**					

3. Stochastic wind and solar power model.

**Fig. 1:**
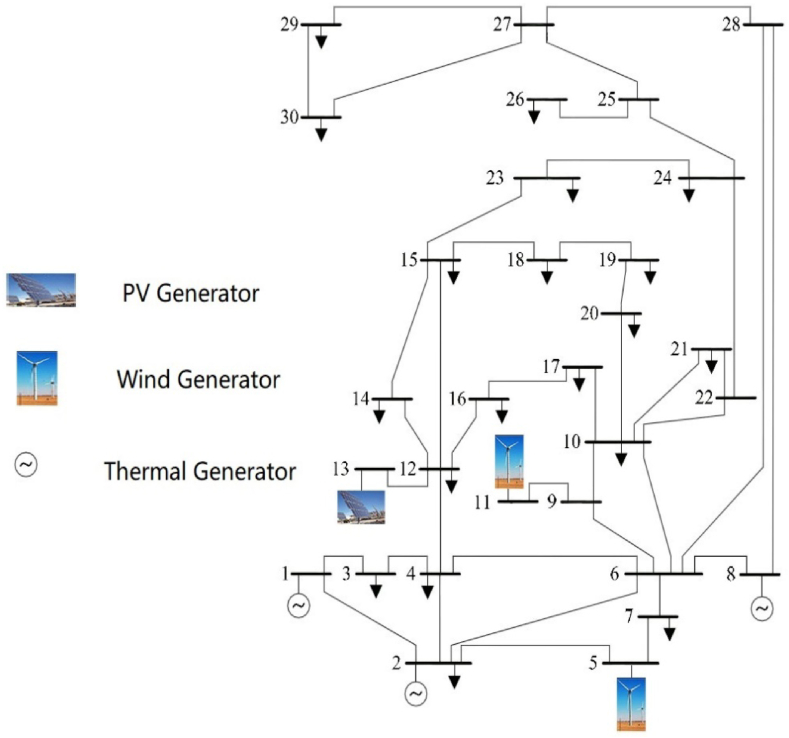
The system architecture diagram of the modified IEEE-30 bus system.

Section 3 is focused to an efficient approach for modeling the renewable energy generators. Replacing the thermal generators with wind turbines and solar PV farms and calculating the cost of two generators. Fig. 1 is a wiring diagram of the modified IEEE-30 bus test system.

### 2.5 Wind power model

In a multitude of simulation experiments, the probability of general wind speed is divided into a skewed normal distribution. With in-depth research, the two-parameter Weibull distribution has been as the better function suitable for the actual wind speed distribution. The Weibull PDF is widely used in wind energy resource assessment and economic evaluation of a wind farm.

#### 2.5.1 Stochastic wind speed probability distribution function

**Fig. 2:**
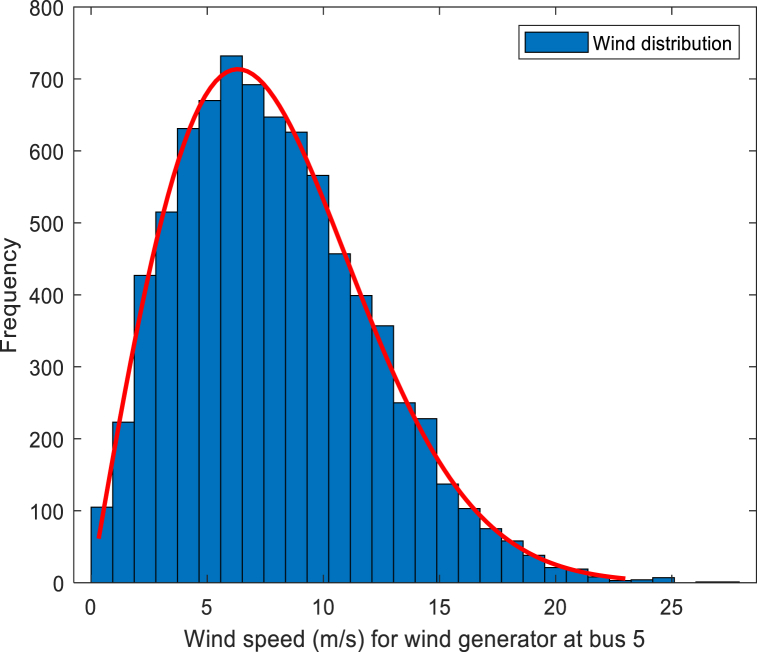
Wind speed fitting curve for wind generator 1.

**Fig. 3:**
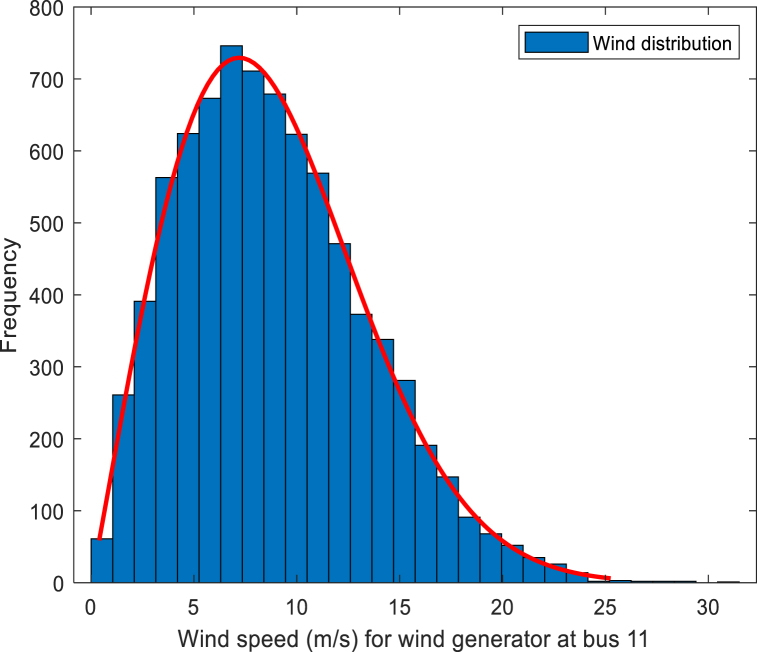
Wind speed fitting curve for wind generator 2.

Referring the two-parameter Weibull distribution, the wind speed PDF is given as Eq. (18):

(18) $$f_{v}\left(v\right)=\left(\frac{k}{\lambda }\right){\left(\frac{k}{\lambda }\right)}^{k-1}e^{-{\left(\frac{v}{\lambda }\right)}^{k}},v\;>0$$

where *v* denotes the actual wind speed, *λ* and *k* denote the scale and shape parameter. Wind speed distribution for wind farms 1 and 2 are shown in Fig. 2, Fig. 3.

In the 30-bus system, two wind generators with different output powers are used. The actual output power (*P*_*w*_(*v*)) of each turbine is calculated as Eq. (19)：

(19) $$P_{w}\left(v\right)=\left\{ \begin{array}{l} 0，\;v<v_{in}\;or\;v>v_{out}\\ P_{\text{wr}}\left(\frac{v-v_{r}}{v_{r}-v_{in}}\right),v_{in}\leq v\leq v_{r}\\ P_{\text{wr}},v_{r}\leq v\leq v_{out} \end{array}\right.$$

where *v*_*in*_ denotes the cut-in wind speed of the turbine, *v*_*r*_ expresses the rated wind speed, and *v*_*out*_ indicates the cut-out wind speed. *P*_*wr*_ indicates the rated power output of the turbine. The specific values of the correlation coefficients are in Table 4.

**Table 4:** Parameters of wind power generator plants.

Windfarm No.	*k*	λ	*v*_*in*_ (m/s)	*v*_*r*_ (m/s)	*v*_*out*_ (m/s)	*g*_*i*_	*K*_*Rw,i*_	*K*_*Pw,i*_
1 (bus 5)	2	9	3	16	25	*g*_*1*_ = 1.60	*K*_*Rw,1*_ = 3	*K*_*Pw,1*_ = 1.5
2 (bus 11)	2	10	3	16	25	*g*_*2*_ = 1.75	*K*_*Rw,2*_ = 3	*K*_*Pw,2*_ = 1.5

The discontinuity, disorder and instability of the wind resource results in variable electrical energy from wind turbines. According to the different cases of wind speed, the probability calculation of three different states is given by Eqs. (20), (21), (22):

(20) $$f_{w}\left\{P_{w}=P_{\text{wr}}\right\}=e^{-{\left(\frac{v_{in}}{\lambda }\right)}^{k}}-e^{-{\left(\frac{v_{out}}{\lambda }\right)}^{k}}$$

(21) $$f_{w}\left\{P_{w}\right\}=\frac{k\left(v_{r}-v_{in}\right)}{\lambda^{k}P_{\text{wr}}}\times {\left[v_{in}+\frac{P_{w}}{P_{\text{wr}}}\left(v_{r}-v_{in}\right)\right]}^{k-1}\times e^{-{\left(\frac{v_{in}+\frac{P_{w}}{P_{\text{wr}}}\left(v_{r}-v_{in}\right)}{\lambda }\right)}^{k}}$$

(22) $$f_{w}\left\{P_{w}=0\right\}=1-e^{-{\left(\frac{v_{in}}{\lambda }\right)}^{k}}+e^{-{\left(\frac{v_{out}}{\lambda }\right)}^{k}}$$

#### 2.5.2 Direct cost evaluation of wind power

Unlike thermal generators, there are no fuel costs for wind generators, but there are costs to purchase wind turbines, which are called direct costs. The direct cost of a wind generator (*C*_*Dw*_) is computed as Eq. (23):

(23) $$C_{\text{Dw}}=\sum_{i=1}^{\text{Nw}}g_{i}P_{\text{ws},i}$$

where *g*_*i*_ expresses the direct cost coefficient, *P*_*ws,i*_ denotes the scheduled power and *Nw* indicates the number of wind generators (*Nw* = 2). The values of the related parameters are provided in Table 4.

#### 2.5.3 Wind power cost evaluation with uncertainties

Due to the discontinuous, disordered and unstable nature of wind energy, wind turbines do not generate electricity continuously, but intermittently, and operators must also consider some other costs. The additional costs that arise in two cases when wind turbines generate excess and deficit power, namely the reserve cost (C_Rw_) and the penalty cost (C_Pw_). The calculation formulas for C_Rw_ and C_Pw_ of a wind power plant are displayed in the following (24), (25):

(24) $$C_{\text{Rw}}=\sum_{i=1}^{\text{Nw}}K_{\text{Rw},i}\int_{0}^{P_{\backslash \text{ws},i}}\left(P_{\text{ws},i}-P_{w,i}\right)f_{w}P_{w,i}d\left(P\left(w,i\right)\right)$$

(25) $$C_{\text{Pw}}=\sum_{i=1}^{\text{Nw}}K_{\text{Pw},i}\int_{P_{\backslash \text{ws},i}}^{P_{\backslash \text{wr},i}}\left(P_{w,i}-P_{\text{ws},i}\right)f_{w}P_{w,i}d\left(P\left(w,i\right)\right)$$

where *K*_*Rw,i*_, *C*_*Rw,i*_ are the reserve and penalty cost coefficients, respectively.

### 2.6 Solar PV power model

Due to the great influence of factors such as the intensity of solar radiation and temperature, the power generation time of PV generators has certain fluctuations and uncertainties. Therefore, in the power grid, energy storage systems will be used for complementary power generation to alleviate the uncertainty of the output of PV generators.

#### 2.6.1 Calculation of solar PV power probabilities

**Fig. 4:**
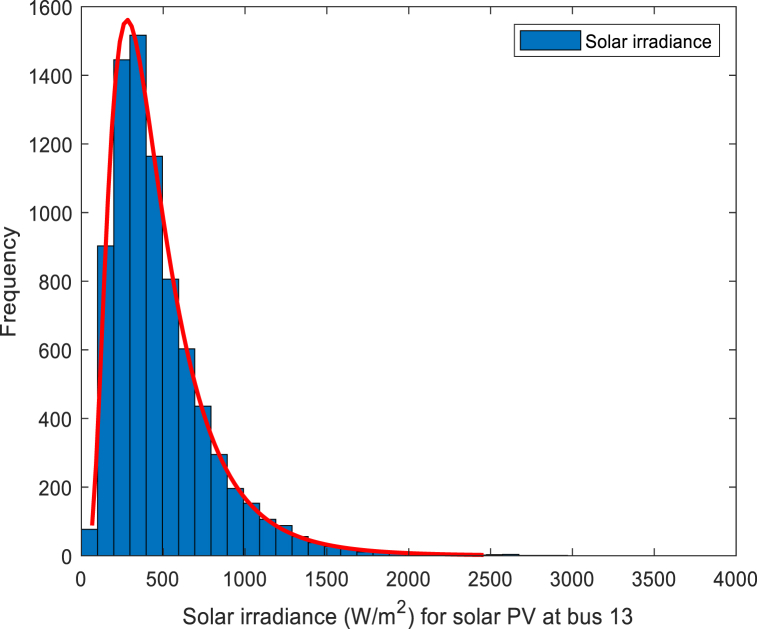
Solar irradiance distribution.

Some studies have pointed out that in a short period (within one day), the illumination of the sun can be approximately regarded as a Beta distribution [19]. Under the premise that the influence of seasonal changes on light intensity is not considered, a large number of papers show that the log-normal PDF can be applied to fit the probability distribution of solar radiation intensity. The log-normal PDF is formulated as Eq. (26):

(26) $$F_{G}\left(G\right)=\frac{1}{G\sigma \sqrt{2\pi }}e^{\frac{-{\left(\ln\;G-\mu \right)}^{2}}{2\sigma^{2}}},G>0$$

where G, σ and μ represent the solar irradiance, standard deviation and mean value, respectively. The values of the coefficients are provided in Table 5. The solar irradiance distribution for solar PV is shown in Fig. 4.

**Table 5:** Parameters of the solar PV plant.

Solar PV plant No.	σ	μ	*G*_*s*_ (W/m^2^)	*G*_*c*_$G_{c}$ (W/m^2^)	*h*_*i*_	*K*_*Rs,i*_	*K*_*Ps,i*_
1 (bus 13)	0.6	6	800	120	*h*_*1*_ = 1.60	*K*_*Rs,1*_ = 3	*K*_*Ps,1*_ = 1.5

**Fig. 5:**
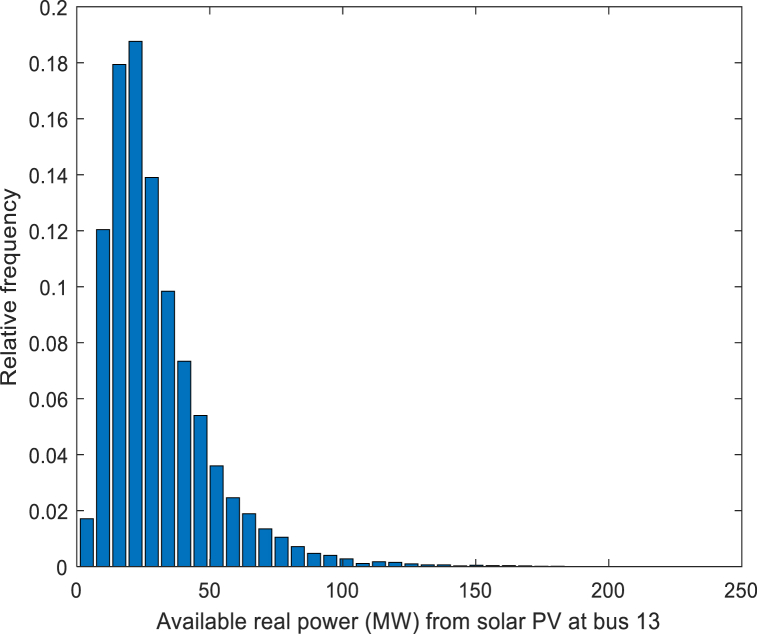
Real power distribution (MW).

In the PV generators, according to Eq. (27), solar irradiance can affect energy conversion with the equation:

(27) $$P_{s}\left(G\right)=\left\{ \begin{array}{l} P_{\text{sr}}\left(\frac{G^{2}}{G_{s}G_{c}}\right),0\;<\;G\;<G_{c}\\ P_{\text{sr}}\left(\frac{G}{G_{s}}\right),G\geq G_{c} \end{array}\right.$$

where $P_{sr}$ denotes the rated output power of the solar PV generator. Fig. 5 is the real power distribution of a solar PV generator.

#### 2.6.2 Direct cost evaluation of solar PV power

According to Ref. [20], the direct cost formula for a solar generator (*C*_*Ds*_) is displayed in the following formula (28):

(28) $$C_{\text{Ds}}=\sum_{j=1}^{\text{Ns}}h_{j}P_{\text{ss},j}$$

where *P*_*ss,j*_ is the scheduled power. *h*_*j*_ represents the direct cost coefficient. *Ns* denote the number of solar PV power plants, in this study *Ns* = 1.

#### 2.6.3 Solar PV power cost evaluation with uncertainties

Similar to wind power generators, storage costs and penalty costs need to be calculated in the solar PV generator. According to Ref. [20], the service cost (*C*_*Rs*_) and penalty cost (*C*_*Ps*_) of solar PV generators are calculated as Eqs. (29), (30):

(29) $$C_{\text{Rs}}=K_{\text{Rs}}\times f_{s}\left(P_{s}<P_{sr}\right)\times \left[P_{sr}-E\left(P_{s}>P_{sr}\right)\right]$$

(30) $$C_{\text{Ps}}=K_{\text{Ps}}\times f_{s}\left(P_{s}<P_{sr}\right)\times \left[E\left(P_{s}>P_{sr}\right)-P_{sr}\right]$$

where *K*_*Rs*_, *K*_*Rs*_ denote the reserve and penalty cost coefficient. Specific coefficient values are provided in Table 5.

## 3 Modified multi-objective Mayfly algorithm

This section will discuss how to solve the MOOPF problem with NSMA-SF. First, the classical MA algorithm is briefly introduced, and an improved MA algorithm (NSMA-SF) is proposed based on it. In NSMA-SF, nonlinear decreasing inertia weights are introduced, and the fast non-dominated sorting in the NSGA-II is referred to. Furthermore, an efficient constraint processing technique, called the superiority of the feasible solutions (SF), is introduced.

### 3.1 Standard mayfly algorithm (MA)

The MA is a new optimization algorithm with an algorithmic mechanism inspired by mayfly populations [21]. Different update behaviors are performed by male and female mayflies. The real world has numerous multi-objective problems, and each objective is often directly conflicting, which cannot be solved by MA algorithms. When the MA algorithm was used to solve the multi-objective optimization problem, the results show that the optimization effect is ineffective. In this case, a better solution can be obtained by compromising each sub-objective.

### 3.2 Multi-objective mayfly algorithm

In the NSMA, as with the standard MA, the initial population was divided into males and females. The two will use different update methods:

(31) $$x_{i}^{t+1}=x_{i}^{t}+v_{i}^{t+1}$$

(32) $$y_{i}^{t+1}=y_{i}^{t}+v_{Fi}^{t+1}$$

Eq. (31) and Eq. (32) are the positional update formulas for male and female mayflies.

#### 3.2.1 Movements of male mayflies

Considering that male mayflies are always performing a nuptial dance, hence they constantly small movements. *pbest*_*i*_ is the individual with the best dominance level obtained so far. The velocity would be updated according to the dominance of *x*_*i*_ and *pbest*_*i*_. The definition of dominance can be seen in Ref. [22].

The corresponding speed update formula is determined according to the dominance of the male mayfly in relation to the *gbest*, as expressed in the following Eqs. (33), (34):

(33) $$v_{ij}^{t+1}=g\cdot v_{ij}^{t}+\alpha_{1}\cdot e^{-\beta r_{p}^{2}}\left(pbest_{ij}-x_{ij}^{t}\right)+\alpha_{2}\cdot e^{-\beta r_{g}^{2}}\left(gbest_{j}-x_{ij}^{t}\right)\;\text{if}\;male\;\text{dominnate}\;gbest$$

(34) $$v_{ij}^{t+1}=g\cdot v_{ij}^{t}+d\cdot rand\;\text{otherwise}$$

where *g* denotes the progressively decreasing gravity coefficient. *a*_*1*_ and *a*_*2*_ are two different attraction constants. *β* denotes a visibility constant, *d* expresses the wedding dance coefficient, and Eq. (35) was used to calculate the Cartesian distance *r*_*p*_ from *x*_*i*_ to *gbest*.

(35) $$\left\|\bar{x_{i}}-\bar{x_{j}}\right\|=\sqrt{\sum_{k=1}^{n}{\left(x_{ik}-x_{jk}\right)}^{2}}$$

#### 3.2.2 Movements of female mayflies

The choice of the velocity update formula for the *i*-th female mayfly *y*_*i*_ is determined by the dominance relationship between *y*_*i*_ and *x*_*i*_. Specifically, if *y*_*i*_ is dominated by *x*_*i*_, the velocity is calculated as Eq. (36):

(36) $$v_{Fij}^{t+1}=g\cdot v_{Fij}^{t}+\alpha_{2}\cdot e^{-\beta r_{\text{mf}}^{2}}\left(x_{ij}^{t}-y_{ij}^{t}\right)$$

where *g* is a gravity coefficient, *r*_*mf*_ represents the Cartesian distance between *x*_*i*_ and *y*_*i*_.

Conversely, if *y*_*i*_ dominates *x*_*i*_, *y*_*i*_ would update the velocity by Eq. (37):

(37) $$v_{Fij}^{t+1}=g\cdot v_{Fij}^{t}+fl\cdot r$$

where *fl* denotes the wandering coefficient.

#### 3.2.3 Mating of mayflies

When female mayflies are attracted to males, they engage in mating behavior. According to random probability, male mayfly *x*_*i*_ and female mayfly *y*_*i*_ are selected to mate and generate two offspring mayflies (*x*_*off1*_ and *x*_*off2*_). The mating behavior can be formulated as Eqs. (38), (39):

(38) $$x_{off1}=G\cdot x+\left(1-G\right)\cdot y$$

(39) $$x_{off2}=\left(1-G\right)\cdot x+G\cdot y$$

where *G* is a random value according to the Gauss distribution.

### 3.3 Performance of fast non-dominated sorting

**Fig. 6:**
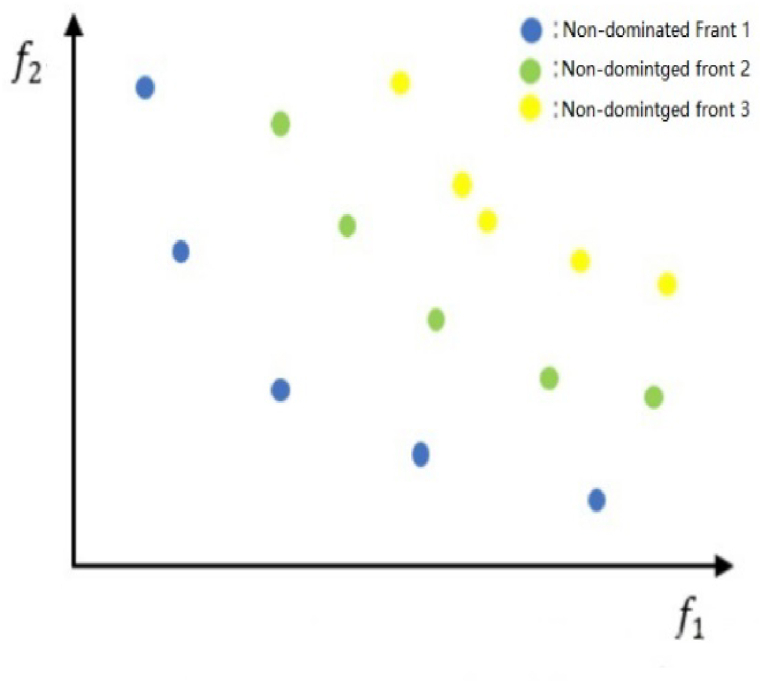
Non-dominant ranking of population.

According to the NSGA-II [22], a population is sorted into different non-dominated levels. Fig. 6 shows a hierarchical schematic diagram of non-dominated ranking. Specifically, each individual solution *s* comes with two additional elements: the total number of solutions dominated by this solution, denoted as *n*_*dom*_; the set of solutions dominated by this solution, denoted as *S*_*p*_. Theoretically, we describe non-dominated sorting will perform uses *O* (*MN*^*2*^) comparisons in the worst case. There are three main steps of the algorithm.

Step 1 Find all individuals *s* with *n*_*dom*_ = 0 and save them in the set *F1*.

Step 2 For each individual *s* in the current set *F1*, find the set *S*_*p*_ dominated by *s*, visit each individual *q* in the set *S*_*p*_ and set *n*_*q*_ = *n*_*q*_-1, if *n*_*q*_ = 0, save the individual *q* in another set *H*.

Step 3 The first non-dominated front is *F1* and set *H* as the initial set.

Repeat the above operations until the entire population being stratified.

### 3.4 Nonlinear decreasing inertia weight

**Fig. 7:**
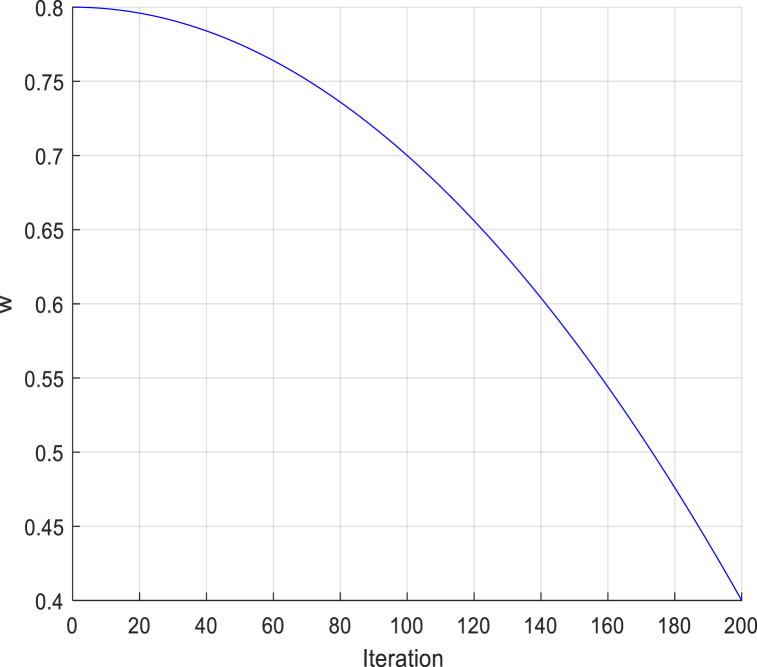
The iteration curve for *w*.

In order to assist in achieving an adequate balance between exploration and exploitation, a nonlinear decreasing inertia weight *w* is introduced. In the early iterations of the NSMA, *w* tends to decrease slowly. The NSMA has a strong global search capability and can find better individuals faster. In the later iterations, *w* tends to decrease rapidly. At this time, the *w* value is smaller, which corresponds to a smaller search range of all the mayflies. The iterative update curve of the value of *w* is shown in Fig. 7. The specific calculation formula is displayed in the following formula (40):

(40) $$w=\bar{w}-\left(\bar{w}-\underline{w}\right)\times {\left(\frac{iter}{\mathrm{Max}\_iter}\right)}^{2}$$

where $\bar{w}\;\text{and}\;\underline{w}$ are the upper and lower bounds of *w*, *iter* and *Max*_*iter* represent the current number of iterations and the predetermined total number of iterations, respectively.

### 3.5 Superiority of feasible solutions (SF)

In [23], SF was mentioned for the first time. Michalewicz and Schoenauer have divided all common methods of constraint handling into five categories [24]. SF is a combination of the 2nd and 3rd of these methods, which is a mixture of the penalty function method and the separation of infeasible solutions to obtain SF. In SF, the equality constraint is first processed, converting equality constraints to inequality constraints using Eq. (41).

(41) $$C_{i}\left(x,y\right)=\left\{ \begin{array}{l} \max\left\{h_{i}\left(x,y\right),0\right\},if\;i\leq H\\ \max\left\{\left|g_{i}\left(x,y\right)\right|-\theta ,0\right\},if\;H\leq i\leq G \end{array}\right.$$

where*θ* identifies a tolerance parameter. *H* is the count sum of inequality constraints and *G* denote the number of total constraints. For any obtained feasible solution *s*_*i*_, *s*_*i*_ must satisfy all inequality constraints *C*_*i*_(*x,y*), including those formed by the transformation of the equality constraints; otherwise, *s*_*i*_ must be an infeasible solution. Next, an entity ε(x,y) is assigned to each infeasible solution $\bar{s_{i}}$ to record the constraint violation degree of $\bar{s_{i}}$. The formula for ε(x,y) can be written as Eq. (42):

(42) $$\varepsilon \left(x,y\right)=\frac{\sum_{i=1}^{G}\left(\varpi_{i}\cdot C_{i}\left(x,y\right)\right)}{\sum_{i=1}^{G}\varpi_{i}}$$

where $\varpi_{i}$ ($\varpi_{i}=1/C_{\max,i}\left(x,y\right)$) represents a weight parameter. Finally, the decision to retain the $\bar{s_{i}}$ is based on the objective function value of the $\bar{s_{i}}$ and the constraint violation degree ε(x,y).

**Fig. 8:**
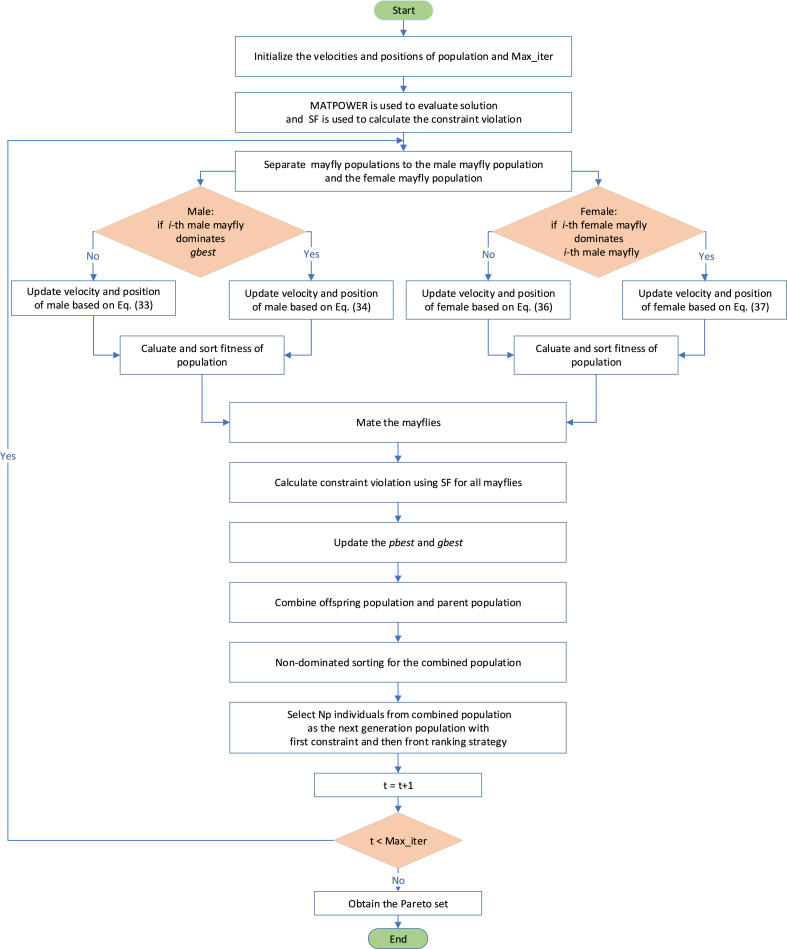
The flow chart for NSMA-SF.

The flow chart of the NSMA-SF for MOOPF problem with stochastic renewable power is presented in Fig. 8.

## 4 Results and comparison

To verify the performance of NSMA-SF, the modified IEEE-30 and standard IEEE-57 bus test system were selected for testing, and ten different cases were conducted. MATPOWER, an open-source tools tool is used to simulate and optimize power systems. All code is done on MATLAB. In addition, the simulated tests have been run on a computer with Intel Core i7 CPU @3.4 GHz and 64 GB RAM. Each study case was run 20 times repeatedly.

The solution to the MOOPF problem is intend to obtain a best compromise solution (BCS) from the best Pareto front (PF) to minimize multiple different and conflicting objectives. The well-known hypervolume (HV) metric is used to access PF [24]. In this experiment, the diversity of PF and the pros and cons of convergence are reflected by comparing the value of HV. The largest one is selected as the best PF, and a set of solutions is selected as the BCS among them.

### 4.1 Results and comparison in the modified IEEE-30 bus system

The system architecture diagram of 30-bus system is presented in the form of a single line diagram, which is shown in Fig. 2. As shown in the figure, it is worth mentioning that two wind-driven generators on buses 5 and 11, and a PV generator is fitted to bus 13. On the system, NSMA-SF is applied to resolve the MOOPF problem with stochastic renewable power. The modified 30-bus system parameter settings were confirmed before the experiment.

As aforesaid, the optimal results obtained by NSMA-SF on six separate cases are reported. First, different cases are analyzed and the values of the load bus voltage and control variables provided by the BCS are verified, and then the BCS calculated by the NSMA-SF algorithm is shown. The obtained BCS is compared with the results in other literature for the MOOPF problem.

#### 4.1.1 Experimental parameter setting

**Fig. 9:**
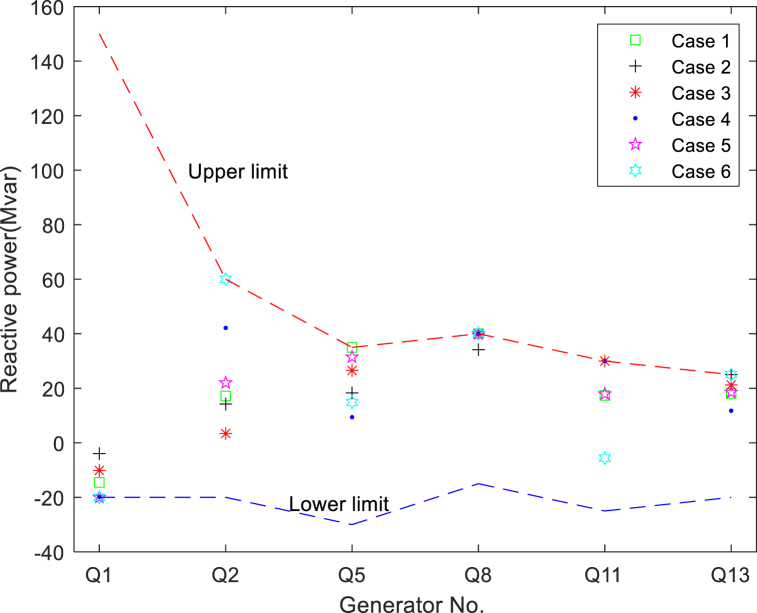
Reactive power of generators in cases 1-6.

A detailed description of the main structural components of the improved IEEE-30 bus test system is listed in Table 6. The allowable limit of each variable is presented in Table 7. These specified values above are consistent with those in other literature. The values for the best single target and BCS for all cases are also listed in Table 8, Table 9. It is obvious from Table 8, Table 9 and Fig. 9 that the constraint variables are all between the set upper and lower limits. The algorithm strictly adheres to the reactive power limit in all cases of the studies conducted in this paper.

**Table 6:** The structural components of the modified IEEE-30 bus system.

Characteristics	Quantity	Detailed description
Buses	30	As seen in [25]
Branches	41	As seen in [25]
Thermal generator	3	Bus no. 1 (swing bus), 2 and 8
Wind-driven generator	2	Bus no. 5 and 11
Solar PV generator	1	Bus no. 13
Control variables	11	The actual power of 5 generators and the voltage of 6 generators, details can be found in Table 7
Connected load	–	283.4 MW, 126.2 MVAr
Load bus voltage	24	Prescribed upper and lower bounds: [0.95–1.05] p.u.

**Table 7:** The maximum and minimum values for each variable in the modified IEEE-30 bus system.

Control variables	Min	Max	State variables	Min	Max
$P_{G_{B2}}\left(\text{MW}\right)$	20	80	$P_{G_{B1}}\left(\text{MW}\right)$	30	140
$P_{G_{B5}}\left(\text{MW}\right)$	0	75	Q_G1_ (MVAr)	−20	150
$P_{G_{B8}}\left(\text{MW}\right)$	10	35	Q_G2_ (MVAr)	−20	60
$P_{G_{B11}}\left(\text{MW}\right)$	0	60	Q_G5_ (MVAr)	−30	35
$P_{G_{B13}}\left(\text{MW}\right)$	0	50	Q_G8_ (MVAr)	−15	40
$V_{B1}\left(p.u.\right)$	0.95	1.10	Q_G11_ (MVAr)	−25	30
$V_{B2}\left(p.u.\right)$	0.95	1.10	Q_G13_ (MVAr)	−20	25
$V_{B5}\left(p.u.\right)$	0.95	1.10			
$V_{B8}\left(p.u.\right)$	0.95	1.10			
$V_{B11}\left(p.u.\right)$	0.95	1.10			
$V_{B13}\left(p.u.\right)$	0.95	1.10

**Table 8:** Simulation results obtained by NSMA-SF.

Case No.	Objectives	Cost ($/h)	Emission(t/h)	VD (p.u.)	Loss (MW)
Case 1	Cost	**782.68**	1.7606	0.53868	5.687
	Emission	903.11	**0.0923**	0.49100	2.2178
	BCS	**861.85**	**0.0975**	0.49609	2.856
Case 2	Cost	**782.58**	1.8274	0.61063	5.6799
	VD	917.06	0.9328	**0.37556**	5.4192
	BCS	**792.63**	1.8016	**0.40204**	6.4101
Case 3	Cost	**782.86**	1.7591	0.43641	5.8161
	Emission	868.19	**0.0962**	0.45634	2.8986
	Loss	874.38	0.09811	0.46611	**2.2386**
	BCS	**856.44**	**0.1100**	0.43785	**2.7762**
Case 4	Cost	**786.00**	1.5524	0.47125	6.0239
	Emission	880.28	**0.0935**	0.96126	2.3234
	VD	901.08	0.16218	**0.39132**	3.6779
	BCS	**859.97**	**0.0981**	**0.70973**	2.8134
Case 5	Cost	**783.47**	1.8383	0.41981	6.0369
	VD	927.38	0.1607	**0.39728**	3.4234
	Loss	866.96	0.1091	0.41714	**1.8449**
	BCS	**801.12**	0.8102	**0.45371**	**4.8642**
Case 6	Cost	**782.81**	1.8001	0.4292	5.9225
	Emission	902.75	**0.0930**	0.64208	2.0771
	VD	809.84	2.4315	**0.39552**	6.4254
	Loss	943.36	0.0954	0.43402	**1.9564**
	BCS	**860.27**	**0.1023**	**0.61484**	**2.638**

*Bold values represent the best values.

**Table 9:** Simulation results of BCSs in study cases 1-6.

Control variables	Case 1	Case 2	Case 3	Case 4	Case 5	Case 6
$P_{G_{B2}}\left(\text{MW}\right)$	51.479	33.913	41.121	51.321	20.79	45.17
$P_{G_{B5}}\left(\text{MW}\right)$	56.686	44.735	71.263	58.377	56.476	70.34
$P_{G_{B8}}\left(\text{MW}\right)$	24.558	15.281	15.001	26.651	10.364	18.074
$P_{G_{B11}}\left(\text{MW}\right)$	53.506	34.015	54.662	47.858	41.862	53.547
$P_{G_{B13}}\left(\text{MW}\right)$	48.632	26.584	39.832	49.213	37.114	41.614
$V_{B1}\left(p.u.\right)$	1.0652	1.0555	1.0599	1.07	1.0572	1.0547
$V_{B2}\left(p.u.\right)$	1.048	1.0569	1.0539	1.049	1.0586	1.0632
$V_{B5}\left(p.u.\right)$	1.0779	0.98946	1.0352	1.0514	1.027	1.051
$V_{B8}\left(p.u.\right)$	1.10	1.0533	1.0389	1.0679	1.0822	1.06
$V_{B11}\left(p.u.\right)$	1.10	1.0957	1.0855	1.10	1.0792	1.081
$V_{B13}\left(p.u.\right)$	1.0495	1.0705	1.0337	1.0929	1.0647	1.082
Objectives						
Cost ($/h)	**861.85**	**792.63**	**856.44**	**859.97**	**801.12**	**860.27**
Emission(t/h)	**0.097533**	1.8016	**0.11004**	**0.098052**	0.81015	**0.10232**
VD(p.u.)	0.49609	**0.40204**	0.43785	**0.70973**	**0.45371**	**0.61484**
Loss(MW)	2.856	6.41	**2.7762**	2.8134	**4.8642**	**2.638**
Other information						
$P_{G_{B1}}\left(\text{MW}\right)$	51.394	135.29	64.298	52.793	121.79	57.43
Worst VD (p.u.)	0.9179	0.6807	0.6214	0.9621	0.7881	0.9033

**Fig. 10:**
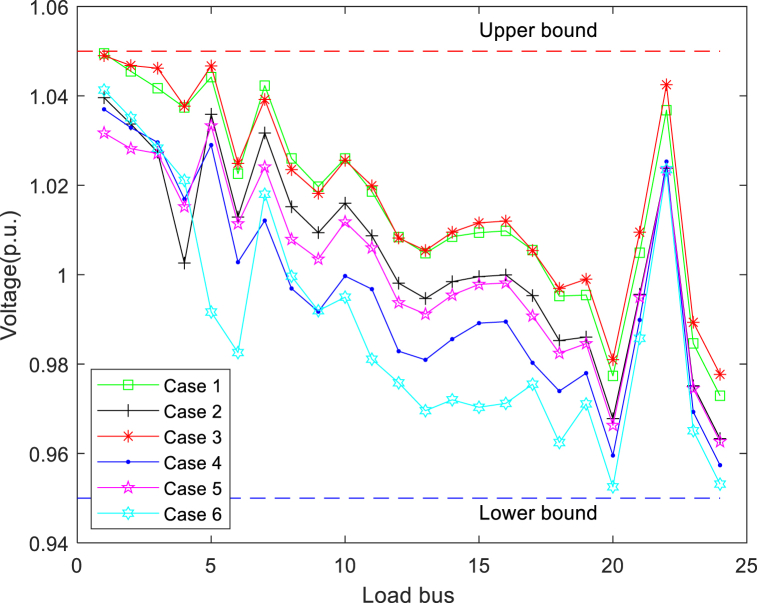
Load bus voltages in cases 1-6.

During the test, the worst total VD values of the load bus are presented in Table 8, Table 9 Presumably, if the voltage limit can also be met with the maximum VD value, the load bus voltage will be safe for all other solutions with smaller VD values. At the highest VD, the distribution of voltage amplitudes on all buses (except for the generator bus) is shown in Fig. 10. It can be seen from the curves that NSMA-SF appropriately satisfies the constraints of the load bus voltage.

#### 4.1.2 Comparative analysis of experiments

Table 8, Table 9 listed the final values of the control variables for the best solutions for a single objective and BCS for six study cases of the modified 30-bus system. From Tables 9 and it is obvious that all 11 control variables are limited within the standard range.

**Fig. 11:**
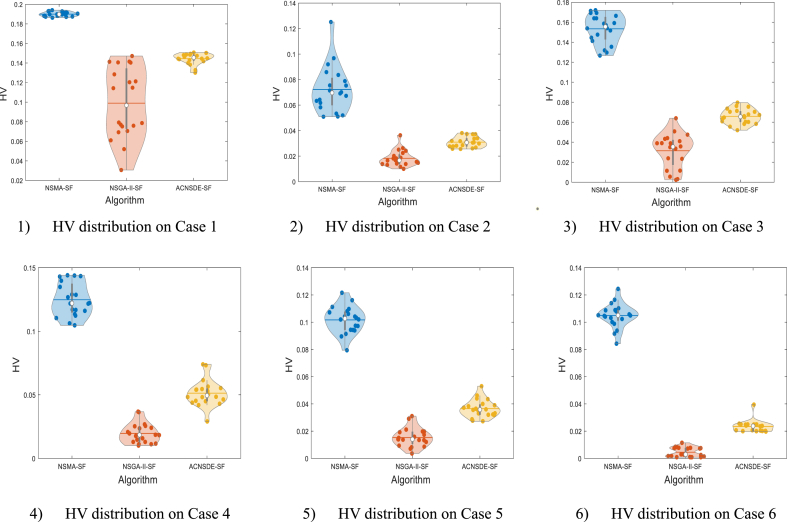
HV values distribution of three algorithms on Cases 1-6.

Table 10 reports the 20 run results of the HV values using NSMA-SF in cases 1–6 and compared with the HV values obtained from NSGA–II–SF [26] and ACNSDE-SF [26]. The result comparison is based on the Max, Min, mean and Std among the 20 independent run times. In addition, nonparametric statistical analysis was performed using the Wilcoxon Signed Ranks test, where *R* + and *R−* refer to the sum of the rankings of NSMA-SF outperforming its competitors or its competitors outperforming NSMA-SF [27]. According to Table 10, the NSMA-SF achieves better HV values in terms of Max, Min and mean compared with two competitive algorithms in all 6 cases. In terms of the Std, the values of NSMA-SF are smaller than those of NSGA–II–SF in cases 2–6, but there is no significant difference between them. Table 10 also lists the 20 runs of NSMA-SF and statistical results of the Wilcoxon rank sum test of two other competitive algorithms for HV values. From the Wilcoxon Signed Ranks test results, in test cases 1–6, the difference in HV values between the proposed NSMA-SF and the other two comparison algorithms is significant. To visually compare the HV value distributions of the three methods, the violin diagram is used to display the distributions. The violin diagram combines the advantages of box and density plots, it can display the distribution state and probability density of multiple sets of data. HV values distribution of three algorithms on cases 1–6 are in Fig. 11. In all 6 cases, the HV value assignment of NSMA-SF was significantly better than NSGA–II–SF and ACNSDE-SF.

**Table 10:** Statistical results of HV indicator value.

Case	Algorithm	HV				Wilcoxon Signed Ranks test			
		Max	Min	Mean	Std	R+	R-	*p*-value	Sig.
Case 1	NSMA-SF	**0.1940**	**0.1863**	**0.1899**	**0.0023**	–	–	–	
	NSGA–II–SF [26]	0.1413	0.0306	0.0986	0.0294	210	0	8.8575E-05	+
	ACNSDE-SF [26]	0.1515	0.1406	0.1462	0.0029	210	0	8.8575E-05	+
Case 2	NSMA-SF	**0.1252**	**0.0509**	**0.07233**	0.0183	–	–	–	
	NSGA–II–SF	0.0263	0.01	0.0183	0.0062	210	0	8.8575E-05	+
	ACNSDE-SF	0.0381	0.0256	0.0312	**0.0041** **.**	210	0	8.8575E-05	+
Case 3	NSMA-SF	**0.1722**	**0.1266**	**0.1534**	0.0144	–	–	–	
	NSGA–II–SF [26]	0.0639	0.0024	0.0314	0.0197	210	0	8.8575E-05	+
	ACNSDE-SF [26]	0.0842	0.0574	0.0686	**0.007**	210	0	8.8575E-05	+
Case 4	NSMA-SF	**0.1441**	**0.1047**	**0.1248**	0.0131	–	–	–	
	NSGA–II–SF [26]	0.0367	0.0037 3	0.0169	**0.0086**	210	0	8.8575E-05	+
	ACNSDE-SF [26]	0.0732	0.029	0.0474	0.0112	210	0	8.8575E-05	+
Case 5	NSMA-SF	**0.1216**	**0.0794**	**0.1017**	0.0098	–	–	–	
	NSGA–II–SF	0.031	0.0036	0.0153	0.0068	210	0	8.8575E-05	+
	ACNSDE-SF	0.0530	0.0273	0.0365	**0.0065**	210	0	8.8575E-05	+
Case 6	NSMA-SF	**0.1245**	**0.0843**	**0.1049**	0.0088	–	–	–	
	NSGA–II–SF [26]	0.0114	0.00089	0.0057	**0.0039**	210	0	8.8575E-05	+
	ACNSDE-SF [26]	0.0394	0.0168	0.0234	0.0054	210	0	8.8575E-05	+

*Bold values represent the best results.

**Fig. 12:**
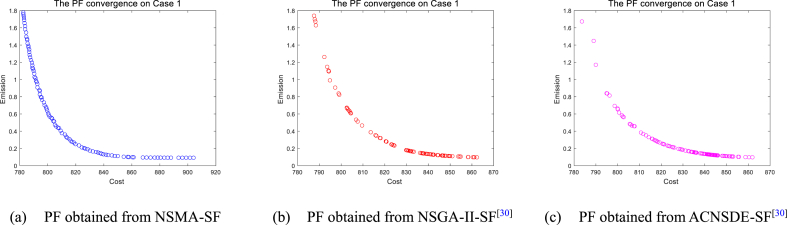
The best PF obtained by three algorithms on Case 1.

**Fig. 13:**
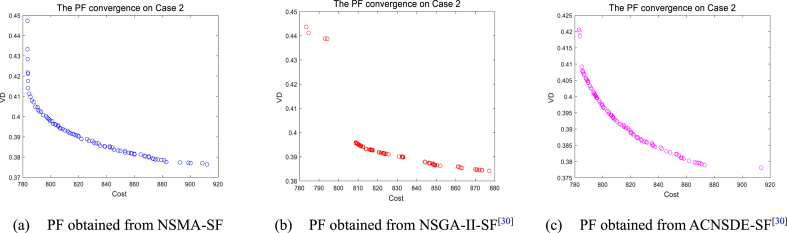
The best PF obtained by three algorithms on Case 2.

**Fig. 14:**
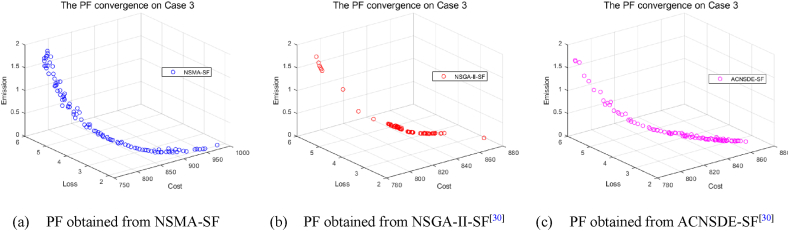
The best PF obtained by three algorithms on Case 3.

**Fig. 15:**
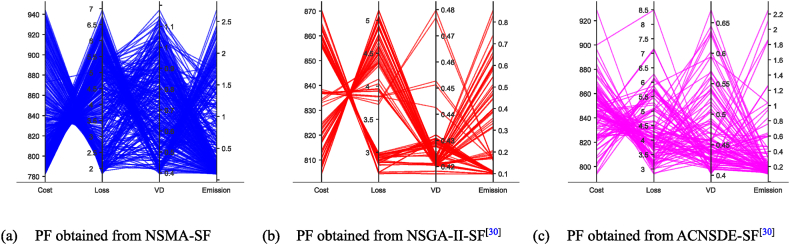
The best PF obtained by three algorithms on Case 6.

It is worth mentioning that the optimal PF is determined by the maximum value of HV, the optimal PF obtained from three algorithms are shown in Fig. 12, Fig. 13, Fig. 14, Fig. 15. The choice of the BCS depends on the distribution of the non-dominated solutions along the PF. The quality of an optimal PF will directly affect the value of the BCS. As shown in Fig. 12, Fig. 13, Case 1 and Case 2 are both 2-objectives optimization. According to the figure, the shape of PF is similar to a curve. It can be seen form Fig. 12, Fig. 13 that the PF of NSMA-SF has a lower aggregation density. In Fig. 12, Fig. 13, NSGA–II–SF and ACDSDE-SF tend to aggregate in local areas. NSGA–II–SF and ACDSEE-SF have poor coverage of true Pareto optimal fronts, so they cannot efficiently find as many global optimal solutions as possible. By comparison, it can be proved that NSMA-SF has better search performance in searching for the optimal PF. Therefore, it is fully proved that NSMA-SF is superior to the other two competitive algorithms in finding multiple Pareto optimal solutions and their corresponding optimal PF in terms of outcome measures in decision space and objective space. The NSMA-SF algorithm ultimately exhibits a very competitive search performance compared to the other two algorithms.

However, as the objective number increases, the difficulty of solving the problem increases exponentially, which results in a significant decrease in the performance of NSMA-SF. For the 3-objectives study cases, cases 3–5, whose true PF shape is a hypersurface, the distribution of PF can be shown in Fig. 14. In case 6, the number of optimization objectives is four, which is difficult to display with a graph. The parallel coordinate plot is used to display the obtained PF. The parallel coordinate plot is suitable for the analysis and comparison of multi-dimensional data (especially when the dimensions are greater than 3), and can directly observe the distribution of each variable. Fig. 15 is a parallel coordinate plot of the BCS of the best PF on case 6. The figure shows a reasonably good distribution of solutions for the four-objective optimization case study using NSMA-SF, and a wider distribution of PFs than those obtained with NSGA–II–SF and ACDSDE-SF.

In other words, the BCS obtained by NSMA-SF has a wider range of choices. In general, NSMA-SF can obtain a diverse and well-distributed PF for all cases. The distribution of PF in the decision space obtained by NSMA-SF is far superior to the results obtained by the other two algorithms. From the above discussion and analysis, the proposed NSMA-SF can provide good coverage of the real PF for the MOOPF problem with stochastic renewable power.

Due to the small amount of literature on integrating renewable energy generators in IEEE-30 bus systems, some results from the literature on solving classical MOOPF problems are selected for comparison. Table 9 presents the BCS for NSMA-SF and other optimization methods and compare the results.

These optimization methods are ACNSDE-SF [26], B-MMOFPA [28], BSA [29], CMICA4 [30], ESDE [10], GWO [31], HHO [32], ICBO [9], ISPEA2 [33], MDE [34], MGBICA [35], MJAYA [36], MOEA/D [8], MOEA/D-SF [37], MOICA [38], MOMICA [38], MOPSO [8], NSGA-II [8], NSGA–II–SF [25], PSO [39], SHADE-SF [20], SMODE-SF [40], SP-DE [6], TLBO [41]. The comparison results are listed in Table 11, Table 12. In Table 9, the BCS obtained by NSMA-SF achieves the best value (minimum value) on one of the multiple optimization objectives. Specifically, simultaneous optimization of cost and emission in case 1, the BCS obtained by NSMA-SF gives the lowest emission value, 0.09753 t/h. In case 2 of cost and VD optimization, the cost objective is the lowest, 792.63 $/h. Papers on MOOPF problem with wind and solar energy with more than two objectives is rare.

**Table 11:** Comparison of BCSs of cases 1–4 for IEEE-30 bus system.

Optimization method		Cost ($/h)		Emission (t/h)	Loss (MW)	VD (p.u.)
**Case1**rowhead						
NSMA-SF^ab^		864.85		**0.097533**	2.856	0.49609
NSGA–II–SF [30]^ab^		856.95		0.101	3.0935	0.4654
TLBO [45]		783.1329		1.8065	5.8742	0.4343
SHADE-SF [23]^ab^		**782.503**		1.762	5.770	0.463
GWO [35]^a^		785.7831		1.7648	6.0324	0.5143
HHO [36]^a^		785.1378		1.3775	6.4716	0.4856
PSO [43]^a^		790.2252		1.7274	6.5713	0.4245
ACNSDE-SF [30]^a^, ^b^		843		0.123	3.5492	0.4356
MOEA/D-SF [41]		829.515		0.2501	–	–
**Case2**rowhead						
NSMA-SF^ab^		**792.63**		1.8016	6.41	0.40204
MOMICA [42]		804.96		–	–	**0.0952**
MOEA/D [8]^b^		799.99		–	–	0.354
MOEA/D-SF [37]	802.406		–		–	0.1362
SP-DE [6]^b^		803.4241		0^b^36424	9.7807	0.09772
ICBO [9]		803.3978		**–**	9.7453	0.1014
BSA [33]		803.4294		0.3546	9.3751	0.1147
**Case 3**rowhead						
NSMA-SF^ab^		856.44		**0.11004**	**2.7762**	0.4378
NSGA–II–SF [30]^ab^		853.54		0.11812	2.8492	0.4401
ACNSDE-SF [30]^ab^		827.33		0.19659	4.1918	0.4365
GWO [35]^a^		832.2049		0^a^0982	2.1056	0.5914
HHO [36]^a^		**811.4170**		0^a^7337	6.8614	0.4006
PSO [43]^a^		898.7527		0^a^0966	2.3776	0.4751
TLBO [45]^a^		882.2742		0^a^1004	2.1891	0.4705
MOPSO [8]		891.48		0.2144	3.9557	–
MOEA/D [8]^b^		902.54		0.2^b^07	3.4594	–
NSGA-II [8]		903.79		0.2103	3.7917	–
MOEA/D-SF [41]		881.012		0.2164	4.1441	–
**Case 4**rowhead						
NSMA-SF^ab^		859.97		**0.09805**	2.8134	0.70973
NSGA–II–SF [30]^ab^		**796.42**		1.3481	6.1125	0.4044
ACNSDE-SF [30]^ab^		850.38		0.11084	3.4526	0.42903
GWO [35]^a^		906.5425		0^a^2085	5.1738	0.3766
HHO [36]^a^		824.4960		0^a^1981	2.5172	0.4572
MOEA/D-SF [41]		842.446		0.2406	–	**0.1092**
TLBO [45]		935.6737		0.2682	5.4925	0.3774
MOPSO [8]		846.93		0.2386	–	0.2188
PSO [43]^a^		843.1084		0^a^3977	5.4799	0.3988
NSGA-II [8]		825.86		0.1421	–	0.2648
MOEA/D [8]^b^		850.28		0.1^b^55	–	0.2332

Bold values represent the best results.

a MOOPF with^a^renewable energy sources.

b MOOPF cons^b^ders the valve point effect in the cost calculation.

**Table 12:** Comparison of BCSs of cases 5 and 6 for IEEE-30 bus system.

Optimization method	Cost ($/h)	Emission (t/h)	Loss (MW)	VD (p.u.)
**Case 5**				
NSMA-SF^ab^	**801.12**	0.81015	**4.8642**	0.45371
MOEA/D [8],^b^	831.81	–	5^b^9923	0.1355
**B-MMOFPA** [28]	843.18	–	5.7886	0.1745
MOEA/D-SF [37]	836.7711	0.25003	5.58223	**0.1258**
MOPSO [8]	827.82	–	6.5929	0.1588
NSGA-II [8]	843.14	–	6.4917	0.1931
**Case 6**				
NSMA-SF^ab^	860.27	**0.10232**	2.638	0.61484
NSGA–II–SF [26],^a^	845.32	0.4^a^792	4.2069	0.39792
ACNSDE-SF [26],^a^	837.46	0.1^a^045	3.6984	0.4179
GWO [31],^a^	**798.1537**	0^a^1958	2.7832	0.4542
HHO [32],^a^	816.7495	0^a^0964	**2.5902**	0.4718
PSO [39],^a^	874.7760	0^a^0976	3.1304	0.6722
TLBO [41],^a^	878.3400	0^a^0958	2.6208	0.4528
MJAYA [36],^a^	837.5116	0^a^2028	4.4866	**0.1117**
SHADE-SF [20]^ab^	810.346	0.891	5.276	0.469
MOEA/D-SF [37]	919.04	0.6221	5.5429	0.453
SMODE-SF [40]	927.049	0.4721	5.3148	0.4215
MOMICA [38]	830.188	0.2523	5.585	0.2978

*Bold values represent the best results.

a MOOPF with^a^renewable energy sources.

b MOOPF cons^b^ders the valve point effect in the cost calculation.

In cases 3 and 4, compared with the BCS obtained by other algorithms, the emission in the BCS of MOOPF with renewable energy is the optimal (minimum). In the 3-objective optimization of case 5, the cost and loss values are the best, but with significantly high level of VD. In case 6, which is optimized with four objectives, the BCS obtained by the proposed NSMA-SF is better than other algorithms in optimizing the emission objective. In case 1, 3, 4 and 6, as long as emission is one of the optimization objectives, the emission of the BCS obtained by NSMA-SF is the best value.

Overall, in all 6 cases, comparing the BCSs for solving the classical MOOPF problem, it can be found that the emissions in the BCSs obtained by the NSMA-SF are far smaller than their corresponding values. Explain that with the incorporation of renewable energy sources in the modified 30-bus test system can significantly reduce emissions. When comparing the optimal results of the simulation with those in other literature, it can be discovered that NSMA-SF can achieve better (smaller) emissions. It shows that NSMA-SF is an effective method for resolving the MOOPF problem with stochastic renewable power.

### 4.2 Results and comparison in the standard IEEE-57 bus system

In order to verify the robustness of the NSMA-SF, a higher-dimensional standard IEEE-57 bus system was chosen to conduct simulation experiments. The details are given in Table 3.

#### 4.2.1 Experimental parameter setting

**Fig. 16:**
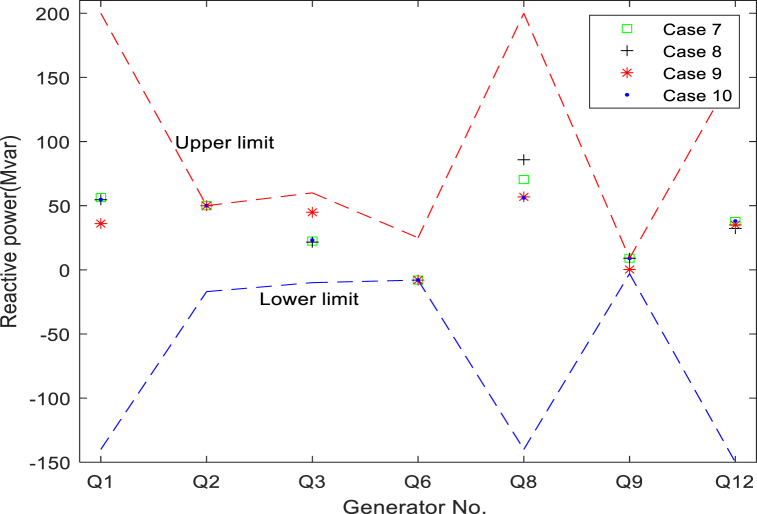
Reactive power of generators in cases 7-10.

**Fig. 17:**
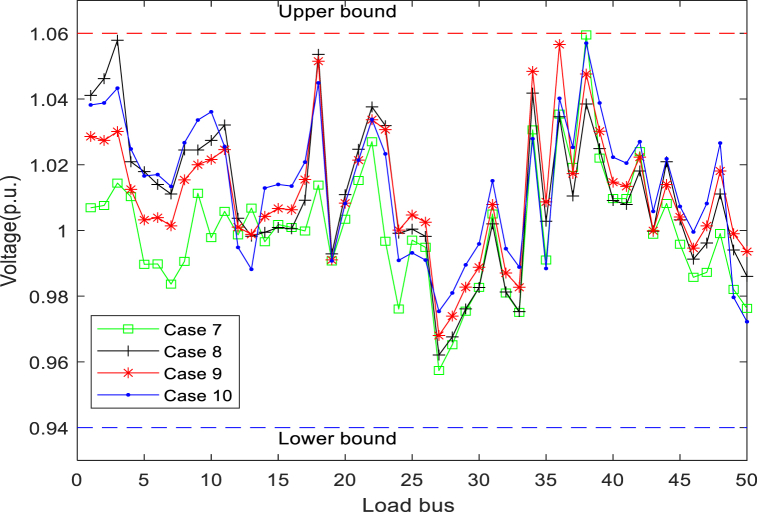
Load bus voltages in cases 7-10.

Table 13 details the composition of the standard 57-bus system. In Table 14, the maximum and minimum values of all variables are presented. Fig. 16 presents the generator reactive power for all cases, showing that the algorithm complies with the generator reactive power constraints in all cases. Fig. 16 suggests that the reactive power of each generator is also strictly controlled within a preset range. The bus load for the four cases with the worst (maximum) cumulative VD of the load bus can be seen in Fig. 17. It can be found from the figure that the VD of each bus is also within the defined upper and lower boundaries. In addition, many load bus voltages were found to operate near the upper limit. This fact proves again that solving the constrained MOOPF problem requires an efficient constraint handling technique.

**Table 13:** The structural components of the standard IEEE-57 bus system.

Characteristics	Quantity	Detailed description
Buses	57	As seen in [41]
Branches	80	As seen in [41]
Thermal generator	7	Bus no. 1 (swing bus), 2, 3, 6, 8, 9 and 12
Shunts	3	Bus no. 18, 25 and 53
Control variables	33	–
Total active and reactive loads	–	1250.8 MW, 336.4 MVAr
Load bus voltage	50	Prescribed upper and lower bounds: [0.94–1.06] p.u.

**Table 14:** The maximum and minimum values for each variable in standard IEEE-57 bus system.

Control variables	Minimum	Maximum	Control variables	Minimum	Maximum
P_G_B2_ (MW)	30	100	T_37_ (p.u.)	0.90	1.10
P_G_B3_ (MW)	40	140	T_41_ (p.u.)	0.90	1.10
P_G_B6_ (MW)	30	100	T_46_ (p.u.)	0.90	1.10
P_G_B8_ (MW)	100	550	T_54_ (p.u.)	0.90	1.10
P_G_B9_ (MW)	30	100	T_58_ (p.u.)	0.90	1.10
P_G_B12_ (MW)	100	410	T_59_ (p.u.)	0.90	1.10
V_B1_(p.u.)	0.95	1.10	T_65_ (p.u.)	0.90	1.10
V_B2_(p.u.)	0.95	1.10	T_66_ (p.u.)	0.90	1.10
V_B3_(p.u.)	0.95	1.10	T_71_ (p.u.)	0.90	1.10
V_B6_(p.u.)	0.95	1.10	T_73_ (p.u.)	0.90	1.10
V_B8_(p.u.)	0.95	1.10	T_76_ (p.u.)	0.90	1.10
V_B9_(p.u.)	0.95	1.10	T_80_ (p.u.)	0.90	1.10
V_B12_(p.u.)	0.95	1.10	State variables		
Q_C18_ (MVAr)	0	20	P_G_B1_ (MW)	0	576
Q_C25_ (MVAr)	0	20	Q_G1_ (MVAr)	−140	200
Q_C53_ (MVAr)	0	20	Q_G2_ (MVAr)	−17	50
T_19_ (p.u.)	0.90	1.10	Q_G3_ (MVAr)	−10	60
T_20_ (p.u.)	0.90	1.10	Q_G6_ (MVAr)	−8	25
T_31_ (p.u.)	0.90	1.10	Q_G8_ (MVAr)	−140	200
T_35_ (p.u.)	0.90	1.10	Q_G9_ (MVAr)	−3	9
T_36_ (p.u.)	0.90	1.10	Q_G11_ (MVAr)	−150	155

#### 4.2.2 Experimental analysis in standard IEEE-57 bus system

**Fig. 18:**
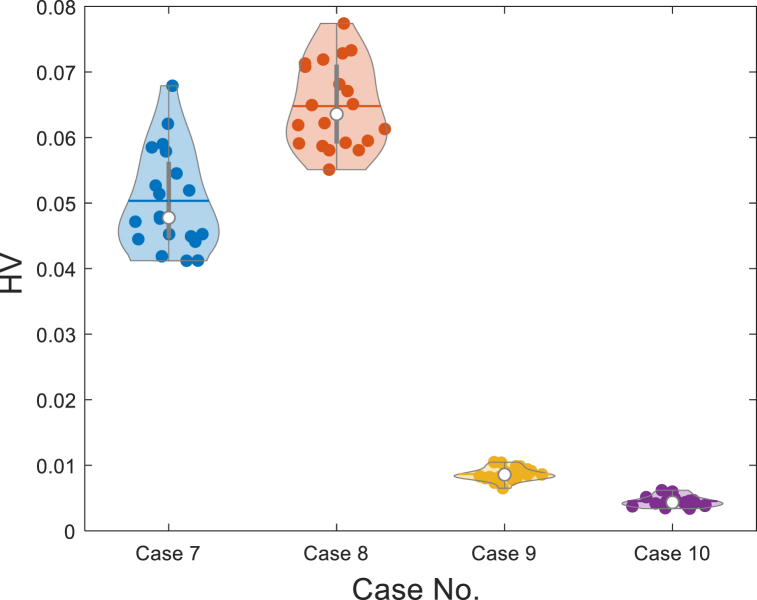
The HV values distribution on cases 7-10.

Table 15, Table 16 list the final values of the control variables for the best solutions for a single objective and BCS for four case studies of the IEEE-57 bus system. Table 17 and Fig. 18 show the HV values and their distributions for cases 7–10, respectively. As the dimension increases, the HV value becomes smaller and smaller. On the other hand, the multi-objective solution increases exponentially due to each additional number of objectives [42]. With the increase in the number of objectives, the HV value decreases significantly, from 0.0648 to 0.0045.

**Table 15:** Simulation results of study cases 7, 8, 9 and 10.

Case No.	Objectives	Cost ($/h)	Emission(t/h)	VD (p.u.)	Loss (MW)
Case 7	Cost	**41 756**	1.5509	1.2404	14.338
	Emission	44 480	**1.1391**	1.1096	15.463
	BCS	**41 786**	**1.5146**	1.1839	13.865
Case 8	Cost	**41 644**	1.9326	2.3523	14.153
	VD	41 737	1.9545	**0.69649**	16.805
	BCS	**41 695**	1.9011	**0.89139**	15.463
Case 9	Cost	**41 902**	1.6398	0.82315	12.538
	Loss	43 462	1.3469	0.74643	**10.99**
	VD	43 274	1.3756	**1.3522**	10.316
	BCS	**42 070**	1.5218	**1.0309**	**11.559**
Case 10	Cost	**42 322**	1.4119	0.95998	12.164
	Emission	43 301	**1.256**	0.81109	12.392
	VD	43 173	1.3702	**0.77072**	11.134
	Loss	43 831	1.3502	1.3033	**10.267**
	BCS	**43 419**	**1.3559**	**1.2405**	**10.413**

*Bold values represent the best values.

**Table 16:** Simulation results of BCSs in study cases 7-10.

Control variables	Case 7	Case 8	Case 9	Case 10	Control variables	Case 7	Case 8	Case 9	Case 10
$P_{G_{B2}}\left(\text{MW}\right)$	99.964	89.538	70.393	56.658	T_B37_ (p.u.)	0.9511	0.9207	0.9074	0.99636
$P_{G_{B3}}\left(\text{MW}\right)$	48.618	44.531	65.502	108.48	T_B41_ (p.u.)	0.9746	0.9760	0.9630	1.0063
$P_{G_{B6}}\left(\text{MW}\right)$	98.484	73.71	92.536	99.535	T_B46_ (p.u.)	0.9862	0.9659	0.9709	0.97521
$P_{G_{B8}}\left(\text{MW}\right)$	404.37	456.2	376.29	316.31	T_B54_ (p.u.)	0.9864	0.9794	0.9745	0.91571
$P_{G_{B9}}\left(\text{MW}\right)$	99.965	99.377	99.93	99.193	T_B58_ (p.u.)	0.9414	0.9277	0.9492	0.98904
$P_{G_{B12}}\left(\text{MW}\right)$	360.51	358.56	408.8	409.6	T_B59_ (p.u.)	0.9659	0.9614	0.9530	0.99241
V_B1_(p.u.)	1.0534	1.0371	1.0445	1.0667	T_B65_ (p.u.)	1.0073	1.0144	0.9944	0.98429
V_B2_(p.u.)	1.0749	1.0779	1.085	1.0638	T_B66_ (p.u.)	1.0261	0.9452	0.9765	0.95843
V_B3_(p.u.)	1.0451	1.0322	1.0416	1.0581	T_B71_ (p.u.)	1.0108	1.011	1.0154	0.9784
V_B6_(p.u.)	1.0101	1.0102	1.0211	1.0022	T_B73_ (p.u.)	1.0918	0.9676	1.0177	1.0044
V_B8_(p.u.)	1.0716	1.0619	1.0615	1.0645	T_B76_ (p.u.)	0.9927	1.055	0.9997	0.99529
V_B9_(p.u.)	1.0632	1.097	1.0562	1.0537	T_B80_ (p.u.)	1.018	0.9930	1.0086	0.99799
V_B12_(p.u.)	1.0424	1.0264	1.0339	1.0453	Objectives				
$Q_{C_{B18}}\left(\text{MVAr}\right)$	10.529	8.1636	8.2047	10.705	Cost	41 786	41 695	42 070	43 419
$Q_{C_{B25}}\left(\text{MVAr}\right)$	11.364	15.955	14.671	14.058	Emission	1.5146	1.9011	1.5218	1.3559
$Q_{C_{B53}}\left(\text{MVAr}\right)$	12.506	13.702	11.900	12.328	VD	1.1839	0.89139	1.0309	1.2405
T_B19_ (p.u.)	1.0252	0.9916	1.0272	0.99068	Loss	13.865	15.463	11.559	10.413
T_B20_ (p.u.)	0.9707	1.0575	1.0084	1.0247	Other information				
T_B31_ (p.u.)	1.0354	1.0124	1.0132	1.0284	$P_{G_{B1}}\left(\text{MW}\right)$	152.75	144.34	148.91	171.44
T_B35_ (p.u.)	1.0033	1.0044	1.0098	0.96743	Worst VD	2.4331	1.954	1.623	1.5246
T_B36_ (p.u.)	0.94868	0.94246	0.94127	1.0227

**Table 17:** The value of the HV indicator obtained by NSMA-SF.

Case No.	HV			
	Max	Min	Mean	Std
7	0.0679	0.0412	0.0503	0.0076
8	**0.0774**	**0.0551**	**0.0648**	0.0064
9	0.0110	0.0065	0.0086	0.0011
10	0.0062	0.0034	0.0045	**0.00084**

**Fig. 19:**
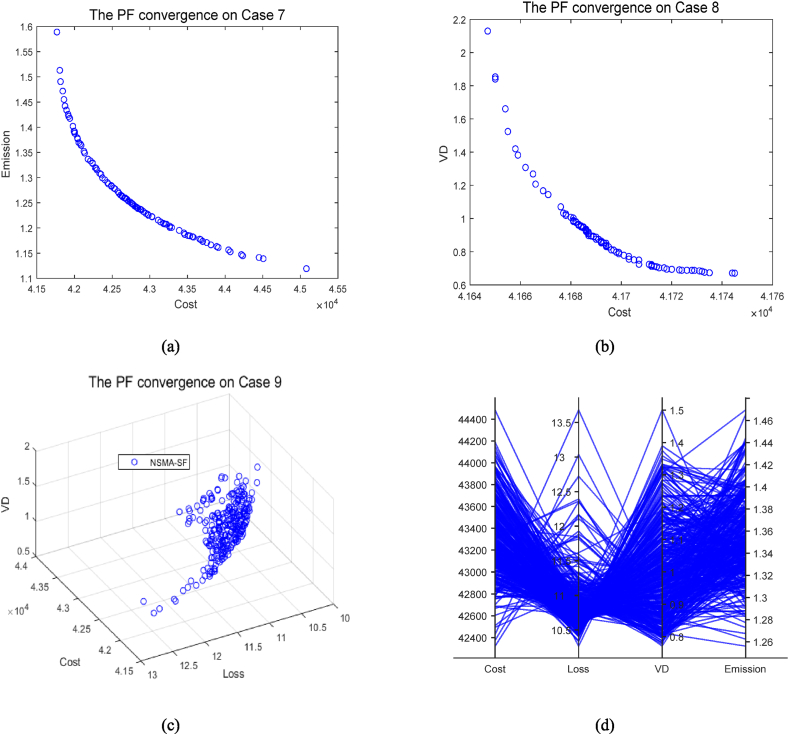
The best PF obtained by NSMA-SF on cases 7-10.

Fig. 19a and b are the optimal PF of cases 7 and 8 for 2-objective optimization, respectively. Fig. 19c is the optimal PF for case 8, where the number of optimization objectives is three. In this case, a PF with reasonably good diversity and distribution is achieved by the NSMA-SF algorithm. For the 4-objective optimization case 10 using NSMA-SF, a good distribution of Pareto solutions is also observed in the parallel coordinates plot in Fig. 19d.

In order to test and verify the ability of NSMA-SF in solving the MOOPF problem, we compare it with other multi-objective algorithms which are used to solve the same problem. Table 18 shows the comparison of the BCS obtained by NSMA-SF and the BCS obtained by other algorithms. It can be seen from Table 18 that no BCS can obtain a best value of every objective in multiple objectives than other algorithms. The BCS obtained by the algorithm does not represent the optimal solution of MOOPF, but it can provide manufacturers with a full reference scheme, so that they can make the final decision according to their own actual conditions. The BCS depends on the strategy and policy of the utility company based on its specific technical and economic forecasts.

**Table 18:** Comparison of BCSs of cases 7,8,9 and 10 for IEEE-57 bus system.

Optimization method	Cost ($/h)	Emission (t/h)	VD (p.u.)	Loss (MW)
**Case 7**				
NSMA-SF^b^	**41786.67**	1^b^5146	1.1839	13.8651
NSGA–II–SF [26],^b^	41 873	1.2^b^32	1.3210	15.2230
ACNSDE-SF [26],^b^	42 504	**1.0**^**b**^**17**	1.2932	12.7642
ESDE [10]	42 863.32	1.2662	–	–
NSGA-II [8]	43 567.77	1.2979	–	–
MOEA/D-SF [37]	42 160.09	1.3183	1.4509	13.0947
ISPEA2 [33]	42 444.55	1.2904	–	–
MGBICA [35]	42 369.07	1.294	–	–
**Case 8**				
NSMA-SF^b^	**41 695**	1.90^b^1	0.89139	15.463
MDE [34]	41 843	–	**0.5962**	–
**Case 9**				
NSMA-SF^b^	**42069.5**	1.^b^218	1.0309	**11.5549**
MDE [34]	42 070	–	**0.6933**	12.4024
CMICA4 [30]	41781.73	–	0.8127	13.9936
NSGA-II [8]	41930.94	–	2.6699	21.5325
MOPSO [8]	41901.36	–	2.0059	16.8022
**Case 10**				
NSMA-SF^b^	43 419	1.35^b^9	1.2405	**10.413**
NSGA–II–SF [26],^b^	43 534	1.2^b^33	0.9293	14.6653
ACNSDE-SF [26],^b^	43 950	**1.0**^**b**^**12**	0.8457	12.9130
MOEA/D-SF [37]	42 648.69	1.3437	**0.6713**	11.886
MOMICA [38]	**41 983.06**	1.496	0.797	13.697
MOICA [38]	41 998.57	1.76	0.87	13.34

*Bold values represent the best results.

b MOOPF cons^b^ders the valve point effect in the cost calculation.

## 5 Conclusion and future work

In this paper, a constraint multi-objective algorithm, namely NSMA-SF is applied to resolve multi-objective OPF with renewable source. Generation cost, power loss, VD, and emission are selected as optimization objectives. The classic IEEE-30 bus system was modified by replacing the three thermal generators connected to it with two wind power plants and one solar photovoltaic power plant. To evaluate the performance of the proposed algorithm, the bus system is used to perform simulation experiments two different PDFs are used to calculate the actual power of uncertainty renewable energy sources. NSMA combined with SF has been successfully solved the MOOPF problem with stochastic wind and solar power. By checking the voltage of each load bus, this paper proves that the NSMA-SF algorithm can effectively. Experimental results show that the NSMA-SF provides a satisfactory BCS in different cases. Specifically, NSMA-SF achieves better PF diversity and Pareto Front (PF) convergence than NSGA–II–SF and ACDSDE-SF for the optimization cases of 2 and 3 objectives. This can also be verified from the distribution of PF. In the case of 4 objectives, NSMA-SF obtains a more uniform and wider distribution PF. From the comparison experiments, the replacement of conventional thermal generators with renewable energy generators has the potential to significantly reduce system pollution emissions and environmental pollution. It is verified that using NSMA-SF to solve the MOOPF problem is an effective approach on a higher dimensional IEEE-57 bus system. What should be pointed out is that the model presented in this paper considers the load of each bus to be deterministic. However, in reality, these bus loads are uncertain. In future work, we suggest that more constraint processing techniques can be used in MOOPF problem with wind and solar energy, such as self-adaptive penalty (SP), epsilon constraint (EC), stochastic ranking (SR) and tested on more complex bus systems.

## Data availability

All data generated or analyzed during this study are included in this published article.

## Additional information

No additional information is available for this paper.

## CRediT authorship contribution statement

**Jianjun Zhu:** Writing – original draft. **Yongquan Zhou:** W. **Yuanfei Wei:** Data curation, Formal analysis. **Qifang Luo:** Investigation, Methodology. **Huajuan Huang:** Software.

## Declaration of competing interest

The authors declare that they have no known competing financial interests or personal relationships that could have appeared to influence the work reported in this paper.
